# Transfer of mRNA Encoding Invariant NKT Cell Receptors Imparts Glycolipid Specific Responses to T Cells and γδT Cells

**DOI:** 10.1371/journal.pone.0131477

**Published:** 2015-06-29

**Authors:** Kanako Shimizu, Jun Shinga, Satoru Yamasaki, Masami Kawamura, Jan Dörrie, Niels Schaft, Yusuke Sato, Tomonori Iyoda, Shin-ichiro Fujii

**Affiliations:** 1 Laboratory for Immunotherapy, RIKEN Center for Integrative Medical Science, Yokohama, Kanagawa, Japan; 2 Department of Dermatology, Universitätsklinikum Erlangen, Erlangen, Germany; University of Southern California, UNITED STATES

## Abstract

Cell-based therapies using genetically engineered lymphocytes expressing antigen-specific T cell receptors (TCRs) hold promise for the treatment of several types of cancers. Almost all studies using this modality have focused on transfer of TCR from CD8 cytotoxic T lymphocytes (CTLs). The transfer of TCR from innate lymphocytes to other lymphocytes has not been studied. In the current study, innate and adaptive lymphocytes were transfected with the human NKT cell-derived TCRα and β chain mRNA (the Vα24 and Vβ11 TCR chains). When primary T cells transfected with NKT cell-derived TCR were subsequently stimulated with the NKT ligand, α-galactosylceramide (α-GalCer), they secreted IFN-γ in a ligand-specific manner. Furthermore when γδT cells were transfected with NKT cell-derived TCR mRNA, they demonstrated enhanced proliferation, IFN-γ production and antitumor effects after α-GalCer stimulation as compared to parental γδT cells. Importantly, NKT cell TCR-transfected γδT cells responded to both NKT cell and γδT cell ligands, rendering them bi-potential innate lymphocytes. Because NKT cell receptors are unique and universal invariant receptors in humans, the TCR chains do not yield mispaired receptors with endogenous TCR α and β chains after the transfection. The transfection of NKT cell TCR has the potential to be a new approach to tumor immunotherapy in patients with various types of cancer.

## Introduction

The use of genetically modified lymphocytes in basic and translational research has increased dramatically in recent years [1, 2]. By engineering CD8^+^ T cells to express TCRs derived from patients’ tumor–specific cytotoxic T cells (CTLs), they can be converted from a population of polyclonal CD8^+^ T cells to CTL of monoclonal TCR specificity [1, 2]. Furthermore, T cells engineered to express MHC-unrestricted chimeric antigen receptors (CARs) have demonstrated efficacy in human trials [1, 2]. These approaches are attractive because CTLs with high affinity and specificity are increasingly easy to generate and can be adapted to treat a number of different tumor types. Almost all of the studies using the TCR gene transfer approach showed T cell response to tumor peptide antigen. However, the innate lymphocyte’s TCR transfer has not been studied. Among innate lymphocytes, invariant natural killer T (NKT) cells have several unique features that differentiate them from T cells and NK cells. NKT cells express a nearly invariant T cell receptor encoded by Vα14Jα18 in mice and Vα24Jα18 in humans and can rapidly produce both IFN-γ and IL-4 after ligand stimulation [3, 4]. An exogenous glycolipid, α-galactosylceramide (α-GalCer), is widely used as a synthetic ligand for activating iNKT cells and is presented to them by the monomorphic, HLA-class I-like molecule, CD1d.

Receptor-transfer strategies using viral vectors, such as retroviral and lentiviral vectors, are often utilized in experiments that require significant transgene expression in primary T cells [1]. The use of viral vector-based gene delivery systems results in stable genomic integration of the transgene and constitutive expression of the transgenic TCR. However, integration of the provirus into the genome may bear the risk of insertional mutagenesis and theoretically, malignant transformation of T cells. Because of this, RNA molecules have recently received attention as a potentially safer delivery system of genomic material to primary lymphocytes. The expression of RNA-derived CAR [5] or RNA-derived TCR [6] in T cells is transient and disappears after short period and a possible toxicity is thought to rapidly abate [5, 7, 8].

γδT cells are innate lymphocytes that comprise 3% to 5% of peripheral blood T cells [9–11]. γδT cells recognize ‘phospho-antigens’, such as isopentenyl pyrophosphate (IPP) and 1-adenosin-5-ylester 3-(3-methylbut-3-enyl) ester (ApppI) via their TCR [9]. The predominant Vγ9Vδ2T subset can be expanded *in vitro* using bisphosphonate zolendronic acid (Zol), which blocks the mevalonate pathway, leading to intracellular accumulation of endogenous γδT cell ligand, IPP and ApppI mevalonate metabolite [9]. Although γδT cell ligands are expressed on some cancer cells and Zol- or bisphosphonate-treated cells, γδT cells in PBMCs of cancer patients often demonstrate impaired activation and proliferation [12]. Different from CTL, γδT cells attack MHC-low expressing tumor cells. Therefore, augmenting γδT cell function in cancer patients could improve patient responses to a broad range of malignancies. To alter the antigen-specificity of γδT cells, an approach to transfect antigen-specific αβTCR genes into γδT cells was recently reported [13, 14]. In the current study, we transfected γδT cells with NKT derived-TCR α- and β-chains and evaluated their anti-tumor effects. This approach resulted in potent bi-functional γδT cells, which recognized both the NKT cell and the γδT cell ligands.

## Materials and Methods

### Human PBMCs and cell lines

Human PBMCs were obtained from healthy volunteers and separated by Ficoll-Hypaque (Amersham Pharmacia Biotech) density centrifugation. All studies were approved by the RIKEN institutional review board and the approval number was [H26-18]. All participants gave written informed consent. Jurkat and K562 cell lines were purchased from RIKEN BRC. HEK293 cell line was purchased from ATCC.

### Reagents and antibodies

IL-2 was purchased from Shionogi & Co., LTD. Human recombinant IL-7 and IL-15 were purchased from Pepro TECH, Inc. α-GalCer and Zoendronic acid (Zol) were purchased from Funakoshi and Novartis Oncology respectively. The following monoclonal antibodies (mAbs) were purchased: anti-human CD11c (B-ly6), CD86 (2311), human invariant NKT Cell (6B11) from BD, anti-human Vα24 (C15), Vβ11 (C21), Vγ9 (IMMU360) from Beckman Coulter, anti-human CD3 (UCHT1) from e-Bioscience and anti-human CD1d (51.1) from Biolegend. Phospho-p44/42 MAPK (Erk1/2) (Thr202/Tyr204) rabbit monoclonal antibody from Cell Signaling and ERK1/2 rabbit polyclonal antibody from Promega were used. HRP-conjugated goat-anti-rabbit IgG was purchased from R&D systems. Laemmli Sample buffer, 2-mercaptoethanol, 10 x Tris/Glycine buffer, 10 x Tris/Glycine/SDS buffer, Any KD Mini-PROTEAN TGX Gel, Immun-Blot PVDF membrane were purchased form BIO-RAD. Immobilon Western Chemiluminescent HRP Substrate was purchased from Millipore. Tris Buffered Saline with Tween20 Tablets, pH 7.6 was purchased from TAKARA. PMA and Ionomycin were purchased from Sigma. The following ELISA kits were purchased: human IFN-γ and IL-4 from BD and human TNF-α from e-Bioscience.

### *In vitro* generation of human NKT cell lines and Vγ9Vδ2 T cells lines

To prepare NKT cell lines, PBMCs were cultured in RPMI 1640 containing 10% FCS the presence of α-GalCer (100 ng/ml) and IL-2 (100 U/ml). After 10–14 days, human NKT cells were sorted using anti-Vα24 mAb and were maintained as NKT cell lines in the presence of IL-2 (100 U/ml), IL-7 (5 ng/ml), and IL-15 (10 ng/ml) in complete medium and restimulated with irradiated PBMCs pulsed with α-GalCer for at least one month as previously described [15].

To prepare Vγ9Vδ2T cell lines, PBMCs were cultured in the presence of Zol (5 μM) and IL-2 (300 U/ml). After sorting using anti-Vγ9 mAb, Vγ9Vδ2T cell cells were maintained as γδT cell line in the presence of IL-2 (300 U/ml).

### Preparation of TCR cDNA and *in vitro* transcription (IVT)

Total RNA was isolated from human NKT cells using RNeasy Mini Kit (QIAGEN Sciences). Complementary DNAs (cDNAs) for NKT-specific invariant TCRα and β chains were amplified by RT-PCR using OneStep RT-PCR Kit (QIAGEN GmbH) and cloned into a bacterial plasmid using QIAGEN PCR Cloning Kit (QIAGEN GmbH). The primer pairs used were as follows: 5’-ATGAAAAAGCATCTGACGACCTTC-3’ and 5’-TCAGCTGGACCACAGCCGCAG-3’ for TCRα; 5’-ATGACTATCAGGCTCCTCTGC-3’ and 5’-TCAGAAATCCTTTCTCTTGACC-3’ for TCRβ. By sequencing analysis, we chose a cDNA clone for each of the TCRs that had a functional open reading frame, and subcloned them into *Eco*RI and *Bam*HI sites of pGEM-3Z vector (Promega). Human NKT-TCR used in this study is as follows, α-chain: TRAV10*01/TRAJ18*01/TRAC*01 (GenBank Accession # DQ341448); β-chain: TRBV25-1*01/TRBD1*01/TRBJ1-4*01/TRBC1*01, with CDR3β sequence being CASEQRGGVDEKL (V region; GenBank #DQ34146), J and C regions; Genbank #BC073930).

RNA was synthesized as described previously [16] with above obtained plasmids that had been linearized with *Bam*HI as templates. Briefly, IVT was carried out using mMESSAGE mMACHINE T7 Ultra Kit (Ambion). This kit utilizes *E*. *coli* poly(A) polymerase to polyadenylate RNA transcripts at their 3’ ends to ensure longer half-life in the cells. The RNA was purified using RNeasy Midi Kit (QIAGEN Sciences) and its integrity was verified by gel electrophoresis.

### Electroporation of *in vitro*-transcribed mRNA

RNA electroporation of T cells was performed as described previously [7]. In brief, PBMCs were stimulated *in vitro* at 10^6^ cells/ml with 50 ng/ml anti-CD3 mAb OKT3 (Janssen pharmaceutical, Inc.) and 300 U/ml IL-2. Three days later, T cells were washed and resuspended in OptiMEM. γδT cells were established as previously described and resuspended in OptiMEM. In transfection by electroporation, 10 μg each RNA was pulsed in square-wave pulse, 500V, 3 msec with an ECM 830 square were electroporation system (BTX). Immediately after electroporation, the cells were cultured with 300 U/ml IL-2 containing culture medium until use in the assay.

### Cytotoxicity assay

The cytotoxic activity against adherent cells was analyzed using an LDH assay kit according to the manufacturer’s instructions (Takara Bio Company). CD1d gene-transfected HEK293 (CD1d-HEK293) were treated with or without 500 ng/ml α-GalCer or 5 μM Zol for 24 h to use them as target cells. Ten thousands target cells were cultured with 1x10^5^ effector cells for 12 h. After subtracting the background control value, cytotoxicity values (%) were calculated as previously described [17].

### Statistical analysis

Differences in *in vitro* data were analyzed using the Student T-test. P<0.05 was considered statistically significant.

## Results

### Cloning of human Vα24^+^Vβ11^+^NKT-TCRα and β DNAs and construction for mRNA synthesis

**Fig 1:**
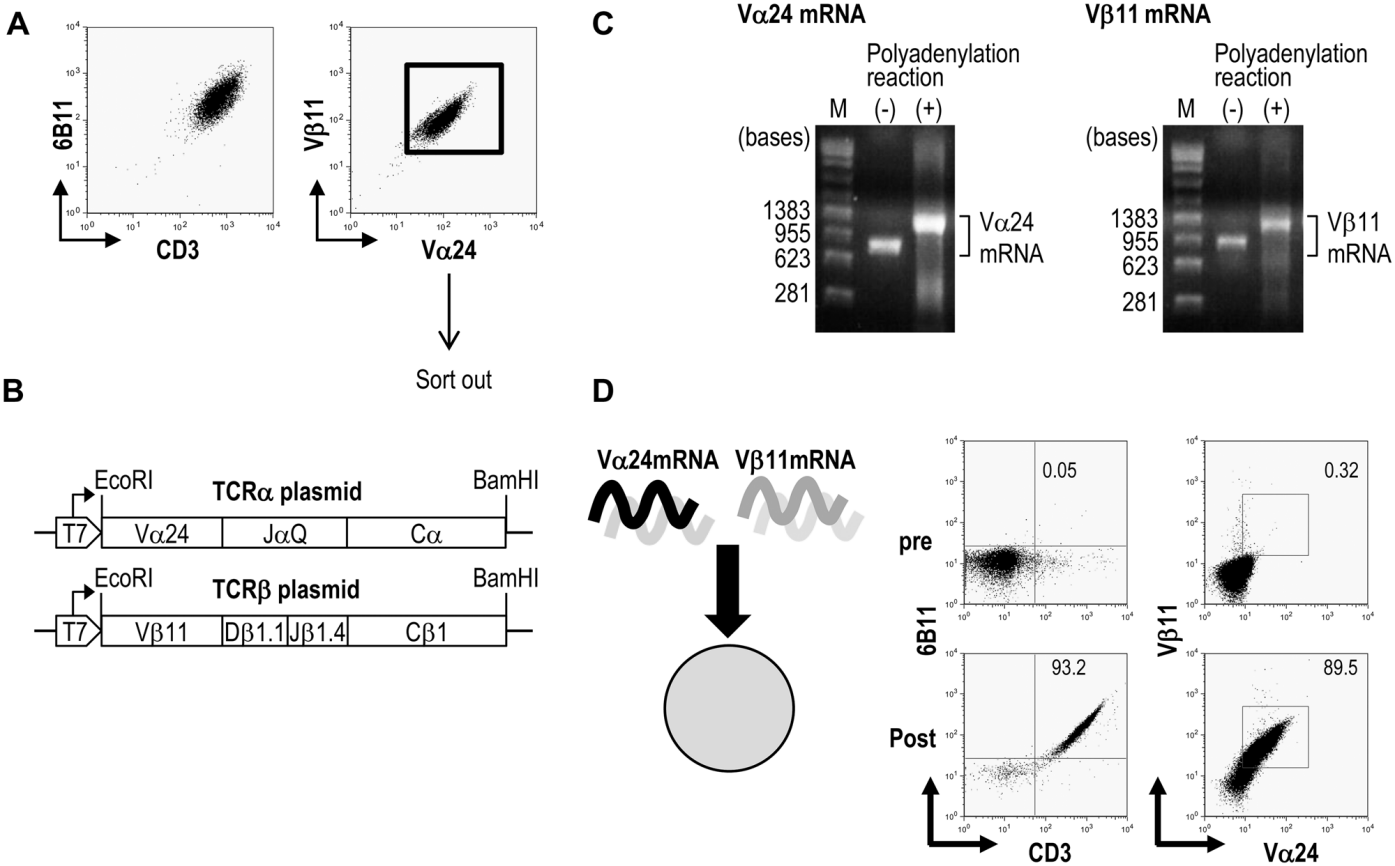
Cloning of human Vα24^+^Vβ11^+^ NKT cell receptor genes and mRNA production. (A) The human Vα24^+^Vβ11^+^ NKT cell line was generated from PBMCs from a healthy donor as described in materials and methods. (B) The NKT cell line was subjected to TCR cloning. The TCR was cloned into the mRNA-production vector pGEM-3Z. Representation of plasmid constructs carrying cDNAs for TCRα (Vα24) and TCRβ (Vβ11) that were used in this study. (T7, T7 promoter on the pGEM-3Z vector; arrows, transcription start site) (C) Representative RNA gel pictures indicating the NKT-TCRα and β chain mRNA after an *in vitro* transcription. TCR mRNAs before and after *in vitro* polyadenylation reaction are shown. mRNAs were electrophoresed on a 1.5% agarose gel. (M, RNA markers) (D) Jurkat cells were electroporated with mRNA of Vα24 and Vβ11 chains of NKT cell TCR. Six hours later, the expression of the NKT-TCR was analyzed with anti-CD3 Ab and anti-6B11 Ab or anti-Vα24 Ab and anti-Vβ11 Ab by flow cytometry. Shown are representative data from at least 4 individual experiments.

We established an NKT cell line from a healthy donor (Fig 1A). After sorting, TCR cDNAs of Vα24^+^Vβ11^+^NKT cells were cloned using RT-PCR. The resultant NKT-TCR α and β pairs, i.e., Vα24 and Vβ11 chains were separately inserted into a pGEM-3Z vector (Fig 1B). We then generated TCR mRNA of Vα24 and Vβ11 using an *in vitro* transcription approach and evaluated that the mRNAs before polyadenylation (Fig 1C (-)) were compatible in size with their template cDNAs whereas the poly-A tails were more than 200 bases long after the reaction (Fig 1C (+)). The expression of NKT-TCR mRNA was tested in the Jurkat T cell leukemic cell line. After transfecting Jurkat cells with both TCR Vα24 and Vβ11 mRNA, the expression of NKT derived-Vα24 and Vβ11 TCR (denoted as NKT-TCR) was confirmed by flow cytometry using anti-Vα24 Ab and Vβ11 Ab (Fig 1D right). In some experiments, we used anti-6B11 Ab which reacts with an epitope of the CDR3 formed by the germ-line configuration of the Vα24 and Jα18 of the TCRα locus of an invariant NKT-TCR (Fig 1D, left). The level of NKT-TCRs was up-regulated in transfected cells after 6 h, and a high expression of Vα24 and Vβ11 TCRs was detected on approximately 90% of Jurkat cells (Fig 1D).

### NKT-TCR mRNA-transfected T cells respond to the NKT ligand

**Fig 2:**
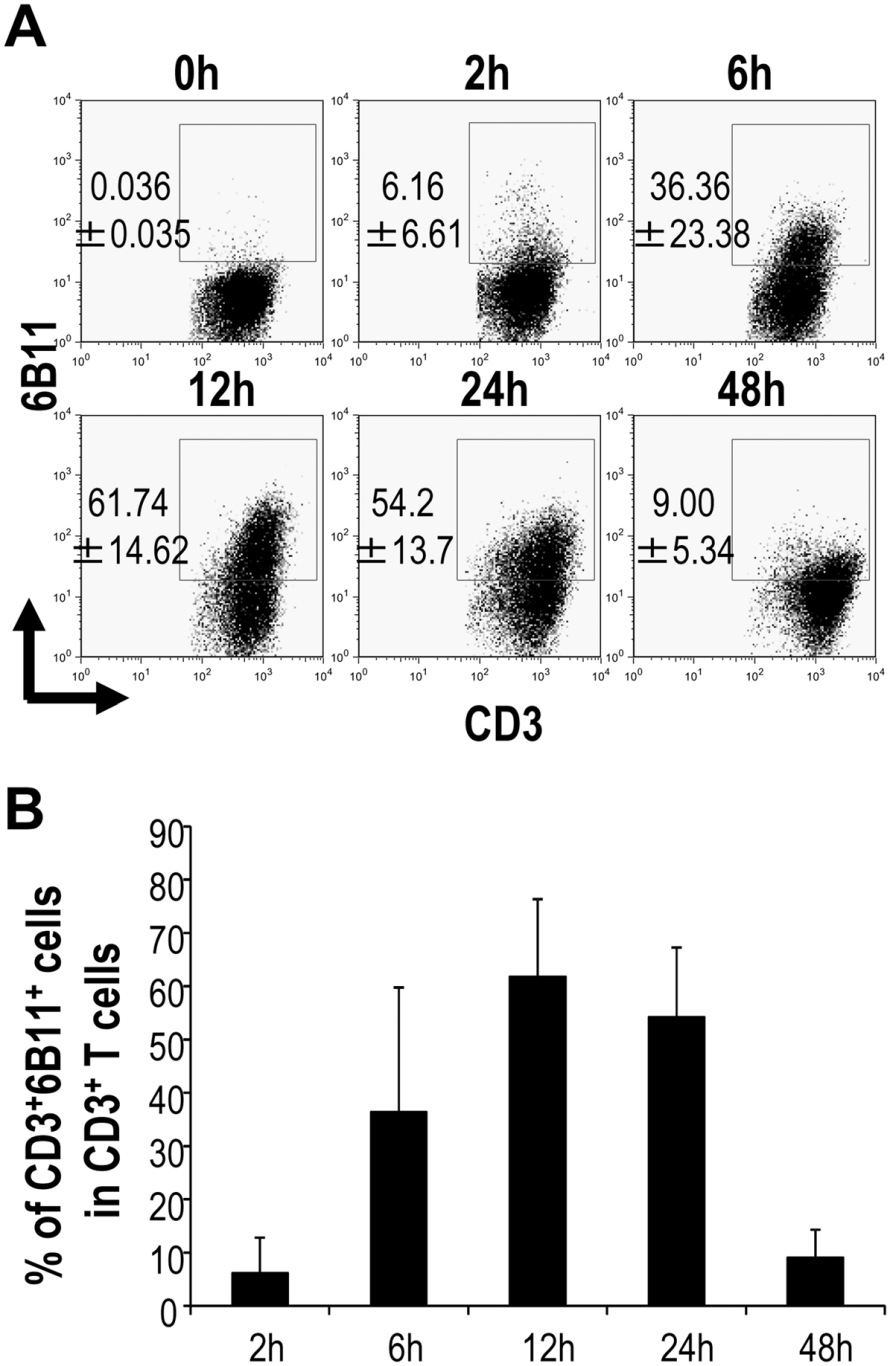
Transfection efficacy of NKT-TCR mRNA into primary T cells. PBMCs were cultured for 3 days in the presence of anti-CD3 and anti-CD28 Ab. Then, they were electroporated with NKT-TCR mRNA. Subsequently, the expression of NKT-TCR was analyzed at indicated times after mRNA electoroporation. The expression of NKT-TCR was analyzed using anti-CD3 and anti-6B11 antibodies. Representative flow cytometry data are shown (A) as well as mean percentages ±SEM from 5 healthy donors (B).

**Fig 3:**
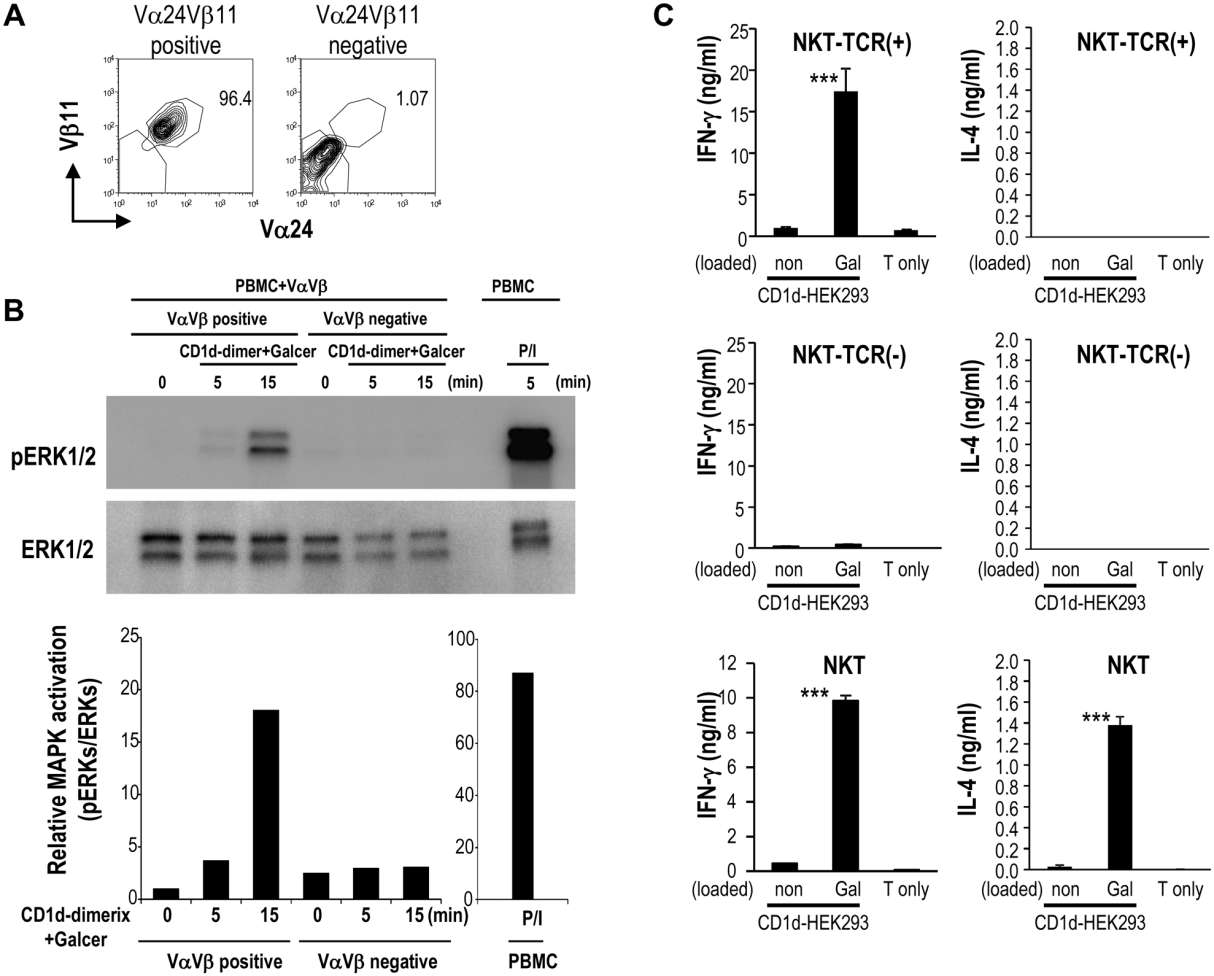
NKT-TCR mRNA transfection confers functionality. As shown in Fig 2, T cells were electroporated with NKT-TCR mRNA. (A) Six hours after NKT-TCR mRNA electroporation, Vα24^+^Vβ11^+^ cells (left panel) and Vα24^-^Vβ11^-^ cells (right panel) were sorted. (B) Each population was exposed to plate-bound CD1d-dimer loaded with α-GalCer for the indicated periods of times. As positive control, PBMCs were activated with PMA (50 ng/ml) and Ionomycin (1 μg/ml) for 5 min. Cells were then lysed and pERK1/2 and ERK1/2 were detected by Western blotting (upper panel). The bar diagram shows a densitometric analysis of the phosphorylated ERK signal from the upper band normalized by the corresponding total ERK signal (lower panel). (C) Sorted 1x10^5^ Vα24^+^Vβ11^+^ cells (top), Vα24^-^Vβ11^-^ cells (middle) or NKT line (bottom) were cocultured with 1x10^4^ CD1d-HEK293 loaded with or without α-GalCer for 24 h. IFN-γ and IL-4 production in the culture supernatants was analyzed by ELISA. Data are mean ±SEM from triplicates and representative of 5 healthy donors with similar results. (***p<0.005 for CD1d-HEK293/Gal vs. CD1d-HEK293/non and T only)

Vα24 and Vβ11 TCR mRNAs were transfected into bulk PBMCs. Expression of NKT-TCR, as measured by flow cytometry started at 2 h, was present on 60–70% of T cells at 12 h, and was still detectable until 48 h (Fig 2A and 2B), confirming that both chains were expressed on the T cells for 48 h. Vα24^+^Vβ11^+^ TCR-transfected cells and Vα24^-^Vβ11^-^ TCR non-transfected cells were sorted using CD3-FITC, Vα24-PE and Vβ11-APC by FACS Aria (Fig 3A). We then investigated whether ERK signaling was induced in a ligand specific way when Vα24^+^Vβ11^+^ TCR-transfected cells were stimulated by the NKT cell ligand. For this purpose, ERK phosphorylation was assessed after stimulation on solid phase of α-GalCer-loaded CD1d-dimer protein. As shown in Fig 3B, Vα24^+^Vβ11^+^ NKT-TCR mRNA-transfected T cells showed the ERK kinase phosphorylation in response to α-GalCer, but not Vα24^-^Vβ11^-^ non-transfected T cells. The MAPK participates in activation of some transcription factors [18, 19]. Indeed, MAPK phosphorylation was detected in NKT-TCR mRNA-transfected T cells when activated by NKT cell ligand, indicating that the transfected TCR indeed recognized the NKT cell ligand (Fig 3B).

NKT-TCR-transfected T cells were then co-cultured with α-GalCer-loaded CD1d-transfected HEK293 cells (CD1d-HEK293/Gal) and IFN-γ secretion in the supernatants was measured. The Vα24^+^Vβ11^+^ NKT-TCR mRNA-transfected T cells (denoted as NKT-TCR(+)) produced high amounts of IFN-γ in an α-GalCer-dependent manner (Fig 3C top, left), but not IL-4 (Fig 3C top, right) whereas NKT cell lines produced IFN-γand IL-4 response to α-GalCer (Fig 3C bottom). However, Vα24^-^Vβ11^-^ TCR non-transfected cells (denoted as NKT-TCR(-)) did not produce IFN-γ (Fig 3C middle, left). Thus, NKT-TCR mRNA transfection resulted in T cells expressing Vα24 and Vβ11 and capable of producing IFN-γ in response to α-GalCer.

### NKT-TCR mRNA-transfected γδT cells respond to both γδT and NKT cell ligands

**Fig 4:**
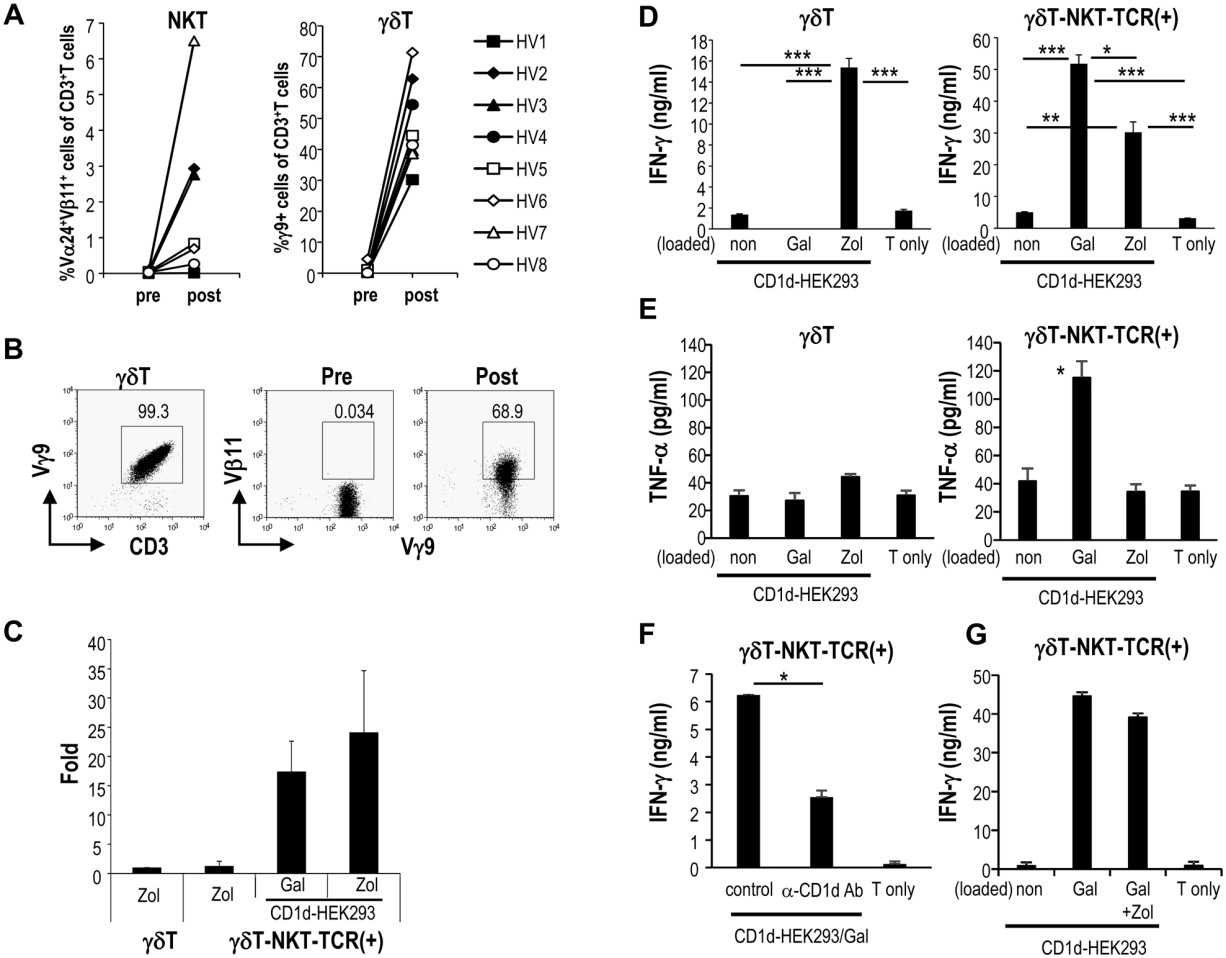
NKT-TCR mRNA-transfected Vγ9Vδ2T cells respond to both the NKT cell ligand and the γδT cell ligand. (A) NKT cells and Vγ9Vδ2T cells were generated from PBMCs. After seven days of culture, the frequencies of NKT and Vγ9Vδ2T cells were analyzed using CD3-APC, Vα24-FITC, and Vβ11-PE or CD3-APC and Vγ9-FITC, respectively by flow cytometry. (HV: healthy volunteer). (B) The mRNAs of Vα24 and Vβ11 TCR chains were transfected into Vγ9Vδ2T cells. Six hours later, the expression of Vβ11 and Vγ9 of pre- and post-electroporation of Vγ9Vδ2T cells was evaluated by flow cytometry. (C) Ex vivo expanded Vγ9Vδ2T cells were transfected with NKT-TCR mRNA, and these cells were cultured again with Zol (5 μM), CD1d-HEK293/Gal or CD1d-HEK293 /Zol. Their number was assessed 3 days later. The data shows fold expansion after stimulation compared with γδT cells stimulated by Zol, which is set as 1. (D, E) Vγ9Vδ2T cells (2x10^5^) were transfected with or without Vα24 and Vβ11 TCR chains (γδT and γδT-NKT-TCR(+)), and IFN-γ (D) and TNF-α (E) production was assessed 48 h after co-culturing with 2x10^4^ CD1d-HEK293/Gal or CD1d-HEK293/Zol. Data are mean ±SEM from triplicates and representative of 5 healthy donors with similar results. (F) As shown in Fig 4D, γδT-NKT-TCR(+) cells were co-cultured with 2x10^4^ CD1d-HEK293/Gal in the presence or absence of anti-CD1d Ab for 6 h. IFN-γ was measured in the supernatants by ELISA. (G) As shown in Fig 4D, γδT-NKT-TCR(+) cells were cocultured with 2x10^4^ CD1d-HEK293/Gal and CD1d-HEK293/Zol (1:1). The supernatants were measured for IFN-γ by ELISA. In F and G, data are mean ±SEM from triplicates and representative of 5 healthy donors with similar results. (*p<0.05, **p<0.01, ***p<0.005)

As shown in Fig 4A, the frequency of NKT cells is lower than that of Vγ9Vδ2T cells in PBMCs of healthy donors (mean±SEM of NKT vs Vγ9Vδ2T of CD3^+^T cells: 0.036±0.035 vs 0.98±1.46). When PBMCs were stimulated with either Zol or α-GalCer, Vγ9Vδ2T cells proliferated more than NKT cells (Fig 4A). Vγ9Vδ2T cells ordinarily do not express NKT-TCR, but transfection of these cells with NKT-TCR mRNA, led to NKT-TCR expression in addition to Vγ9Vδ2T (Fig 4B). To test the functionality of Vα24^+^Vβ11^+^ NKT-TCR mRNA-transfected Vγ9Vδ2T cells (denoted as γδT-NKT-TCR (+)), these cells were stimulated using Zol and α-GalCer. It is well known that Vγ9Vδ2T cells in PBMC can be expanded after culturing with Zol. However, there are few reports about secondary expansion of γδT cells. It was previously reported that the proliferative response of γδT cells is transient and also that repeated injection of BrHPP and IL-2 induced activation induced cell death of Vγ9Vδ2T cell and an exhaustion of the response [20–22]. After *ex vivo* expansion from PBMCs using Zol, we assessed the number and function of γδT cells in secondary challenge with antigen. Vγ9Vδ2T cells were cultured again in the presence of soluble Zol to serve as a control for the subsequent groups (Fig 4C). The number of NKT-TCR-transfected Vγ9Vδ2T cells was higher after stimulation with CD1d-HEK293/Gal cells and CD1d-HEK293/Zol than that of parental γδT cells in secondary challenge (Fig 4C).

We evaluated whether transfection of Vα24^+^Vβ11^+^ NKT-TCR mRNA into Vγ9Vδ2T cells allowed for activation by the NKT cell ligand. Vγ9Vδ2T cells secreted IFN-γ and low level of TNF-α after culturing with CD1d-HEK293/Zol cells, but not after the culture with CD1d-HEK293/Gal cells (Fig 4D and 4E, left). NKT-TCR-transfected Vγ9Vδ2T cells, in contrast, produced more IFN-γ and TNF-α in response to CD1d-HEK293/Gal than CD1d-HEK293/Zol. These data showed that NKT-TCR transfected Vγ9Vδ2T cells responded to NKT cell and γδT cell ligands (Fig 4D and 4E, right). To evaluate whether the activation of NKT-TCR transfected γδT cells is dependent on CD1d molecule, we cultured the cells with a CD1d blocking antibody. As shown in Fig 4F, activation of NKT TCR-γδT cells by α-GalCer is dependent on CD1d. Finally, we examined whether NKT-TCR transfected γδT cells can respond synergistically to both α-GalCer and γδT ligands. As shown in Fig 4G, IFN-γ production by NKT TCR-γδT cells after stimulation with both α-GalCer-loaded CD1d-HEK293 cells and Zol-treated CD1d-HEK293 cells are almost equal to that by NKT TCR-γδT cells by α-GalCer-loaded CD1d-HEK293 cells.

### NKT-TCR mRNA-transfected γδT cells show cytotoxicity against glycolipid-expressing target cells and K562 cells

**Fig 5:**
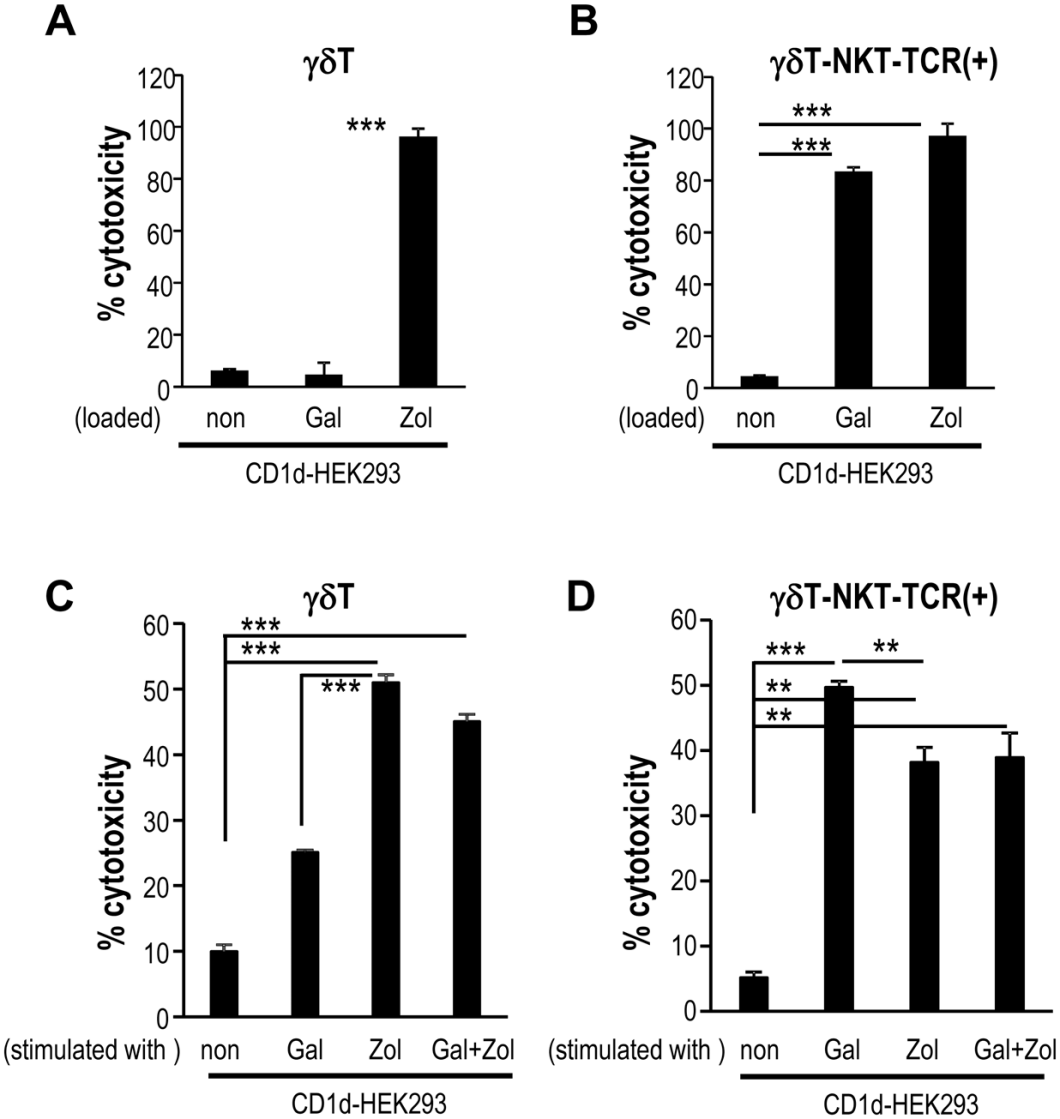
NKT-TCR-transfected γδT cells show cytotoxicity against glycolipid-expressing target cells and K562 cells. (A, B) To test the lysis of ligand-loaded target cells, the cytotoxic activity of γδT (A) or γδT-NKT-TCR(+) cells (B) against CD1d-HEK293, CD1d-HEK293/Gal, CD1d-HEK293/Zol was assessed by LDH assay at an E:T ratio of 10. (C, D) γδT (C) or γδT-NKT-TCR(+) cells (E) were stimulated by CD1d-HEK293/Gal, CD1d-HEK293/Zol or both and then assessed for cytotoxicity against K562. Data are mean ±SEM from triplicates and representative of 4 healthy donors with similar results. (**p<0.01, ***p<0.005)

We and others showed that NKT cells can show cytotoxicity against α-GalCer-loaded CD1d-expressing target cells, such as B16 melanoma, EL4 lymphoma, WHEI3B leukemia and NIH3T3 fibroblasts [16, 23]. Furthermore, γδT cells are cytotoxic against Zol-treated target cells [24]. As shown in Fig 5A, Vγ9Vδ2T cells lysed CD1d-HEK293/Zol cells, but not CD1d-HEK293/Gal cells (Fig 5A). We evaluated whether NKT-TCR-transfected Vγ9Vδ2T cells can show cytotoxicity against target cells loaded with either ligand. They showed high cytotoxicity against CD1d-HEK293/Zol cells as well as CD1d-HEK293/Gal cells (Fig 5B).

Next, the cytotoxicity of NKT-TCR-transfected Vγ9Vδ2T cells against tumor cells was assessed. It is known that Daudi cells (a Burkitt’s lymphoma cell line) are a highly sensitive γδT cell target due to the expression of phosphoantigen while K562 cells (myelogenous leukemia cell line) are a weakly sensitive γδT cell target [10]. We initially confirmed that Daudi cells are killed by γδT cells (40–70% of cytotoxicity, E/T = 10, data not shown), but K562 are not (Fig 5C). Therefore, we used K562 to evaluate antitumor effect in this study. Although γδT cells without stimulation did not show cytotoxicity against K562, they showed cytotoxicity against K562 after stimulation with CD1d-HEK293/Zol (Fig 5C). Then, we measured the NKT-TCR-transfected Vγ9Vδ2T cell-mediated cytotoxicity against K562. NKT TCR-transfected Vγ9Vδ2T cells without culturing did not show cytotoxicity against K562 (Fig 5D). However, after stimulation with CD1d-HEK293/Zol or CD1d-HEK293/Gal, NKT-TCR-transfected Vγ9Vδ2T cells demonstrated a strong killing activity against K562 compared to unstimulated one (Fig 5D). Since K562 did not express CD1d (data not shown), the cytotoxic activity of NKT-TCR-transfected Vγ9Vδ2T cells was not in an NKT TCR-mediated manner. NKT-TCR-transfected Vγ9Vδ2T cells activated by CD1d-HEK293/Gal demonstrated higher cytotoxicity against K562 than those were activated by CD1d-HEK293/Zol. Next, we tested whether NKT-TCR-transfected Vγ9Vδ2T cells can be activated synergistically by both CD1d-HEK293/Zol and CD1d-HEK293/Gal. When NKT-TCR-transfected Vγ9Vδ2T cells were activated by CD1d-HEK293/Gal together with CD1d-HEK293/Zol, the cytotoxicity was similar to that by CD1d-HEK293/Gal (Fig 5D). Thus, NKT-TCR mRNA-transfected Vγ9Vδ2T cells can be primed with α-GalCer and then respond to γδT ligands in a more functional fashion than parental γδT cells.

## Discussion

The current study evaluates the effects of transfecting human NKT cell-derived TCR α and β chain mRNA into either innate or adaptive lymphocytes. NKT-TCR transfected T cells and γδT cells secreted IFN-γ in a ligand-specific manner. These γδT cells demonstrated cytotoxicity against K562 tumor cells and proliferated in response to NKT cell and γδT cell ligands. This study demonstrated that γδT cells and primary adaptive lymphocytes transfected with the NKT-TCR retain parental T cell qualities while acquiring NKT cell functionality, thus creating a bi-potential T cell.

NKT cell receptors are universal in humans and their specificity is not HLA restricted. Therefore, the approach of NKT-TCR mRNA transfection into primary lymphocytes and γδT cells has a number of unique features that makes it attractive for use as an immunotherapeutic approach to cancer treatment. NKT-TCR mRNA is not stably integrated into the genome [5, 6] and is only expressed by transfected cells for a limited period of time, thus eliminating potential safety concerns. On the other hand, Zhao et al demonstrated that CAR mRNA was useful and safer as well, but the retroviral transfer of CAR DNA to T cells was effective for a long time [5]. As a future plan, we will examine about the efficacy using T or γδT cells retrovirally transduced with the NKT-TCR DNA.

A potential disadvantage of the TCR gene transfer approach is the possible formation of mixed TCR dimers. It has been reported that chains of the introduced TCR can pair with endogenous TCR chains, resulting in a dimer composed of both endogenous TCR and introduced TCR, thus altering TCR specificity [13]. However, since γδT cells do not have αβ chains, transfection of NKT α and β chains into γδT cells will not result in mispaired receptors.

NKT cells in naïve mice have the potential to produce both IFN-γ and IL-4 in response to α-GalCer, therefore modulating whether the immune response is Th1 or Th2 [3]. However, NKT cell secretion of IFN-γ, and not IL-4 after an administration of α-GalCer-loaded DCs has been shown to have tumor protective effects [25]. This is further demonstrated in patients with advanced non-small cell lung cancer in whom increased numbers of IFN-γ-producing PBMCs correlated with prolonged median survival time upon an administration of α-GalCer pulsed IL-2/GM-cultured PBMC [26].

In the current study, NKT-TCR-transfected lymphocytes responded to NKT cell ligand with the secretion of IFN-γ, but not IL-4. We found that the transfer of mRNA encoding NKT cell receptors drives the other lymphocytes toward IFN-γ producing NKT like cells, which might be useful against tumor cells. One of the reasons is that the transfected lymphocytes might be already polarized or skewed toward Th1 type in cytokine production. Previous studies showed that PBMCs stimulated by anti-CD3 plus IL-2 led to T-bet via TCR signaling pathway for IFN-γ in most of conventional T cells [27]. T-bet is known to be important in that it regulates antigen-driven effector T cells [28]. Although Zhao et al described a minor population of CD4T cells capable of producing IL-4 or IL-10 in these anti-CD3 Ab and IL-2 stimulated PBMCs [29], major population which were polarized to Th1 might mask the response of Th2. But, further study is needed to understand the mechanism in detail.

Aminobisphosphonates, such as palindronate- or zoledronic acid-activated Vγ9Vδ2T cells to produce IFN-γ and exhibit cytotoxicity against some tumor cell types, e.g. Daudi, K562, AML cells, lymphoma cells and myeloma cells [30–32]. Dieli et al showed that administration of zoledronic acid induces Vγ9Vδ2T cells to mature toward an IFN-γ-producing effector phenotypes [33]. In a recent lung cancer study, adoptive transfer of *ex vivo* expanded Vγ9Vδ2T cells showed some clinical efficacy [11, 34]. The current study demonstrated that NKT-TCR-transfected Vγ9Vδ2T cells can be stimulated and functionally activated by either of the two ligands. These results offer a potentially new treatment modality for activating poorly responding γδT cells in cancer patients with NKT cells ligand for improved tumor responses.

The current study demonstrated that the transfer of NKT-TCR αβ chains enhances the function of both innate and adaptive lymphocytes and this approach may hold the promise of a new anti-cancer immunotherapy.
